# Relative Age Effect in Physical Fitness during the Elementary School Years

**DOI:** 10.3390/pediatric13020040

**Published:** 2021-06-08

**Authors:** Clemens Drenowatz, Gerson Ferrari, Klaus Greier, Franz Hinterkörner

**Affiliations:** 1 Division of Sport, Physical Activity and Health, University of Education Upper Austria, 4020 Linz, Austria; 2 Escuela de Ciencias de la Actividad Física, el Deporte y la Salud, Universidad de Santiago de Chile (USACH), Santiago 7500618, Chile; gersonferrari08@yahoo.com.br; 3 Division of Physical Education, Private Educational College (KPH-ES), 6422 Stams, Austria; nikolaus.greier@kph-es.at; 4 Department of Sports Science, Leopold-Franzens University Innsbruck, 6020 Innsbruck, Austria; 5 Olympic Training Center Upper Austria, 4020 Linz, Austria; franz.hinterkoerner@ooe.gv.at

**Keywords:** children, cardiorespiratory endurance, muscular strength, muscular power, speed, agility, object control, biological maturation

## Abstract

Age-groups are commonly implemented in education and sports in order to provide fair and equal opportunities. Various studies, however, have shown a competitive advantage for early born children over their relatively younger peers, which is referred to as relative age effect. The present study examined differences in various components of physical fitness in Austrian elementary-school children. A total of 18,168 children (51% boys) between 6 and 11 years of age provided valid data on anthropometric characteristics and physical fitness. Specifically, children completed eight fitness tests that assessed cardiorespiratory endurance, muscular strength and power, speed, agility, flexibility and object control. Across age-specific quartiles, older children were significantly taller and heavier than their younger peers. Older children also displayed better performance for strength and power, speed, agility and object control, while differences in cardio-respiratory endurance were less pronounced. These results highlight the presence of a relative age effect during the elementary school years and emphasize the need to consider individual differences in the evaluation of children’s performance. As all children should be given equal opportunities to engage successfully in physical education and sports, physical education teachers and youth coaches need to be educated on the implications of a relative age effect.

## 1. Introduction

Children are commonly grouped by chronological age in various fields, including education, with the purpose of providing fair opportunities and ensuring an adequate and uniform educational process [1]. The grouping of children and adolescents by chronological age, however, still produces a mix of older and younger individuals. The enrollment of children into school grades by birth date, for example, results in an age difference of up to 12-months within a single grade. This difference in chronological age between subjects within a specific age group is referred to as relative age difference [2,3]. Given the differences in maturation and development along with the interaction of genetic factors and experience, the relative age difference can provide advantages and disadvantages for an individual in school and sports performance [4,5]. The potential consequences in specific contexts or domains due to differences in birth date within a given age group are referred to as relative age effect (RAE) [3,4].

The RAE has been initially documented in the context of education [6,7,8] but is also well-established in competitive sports [4,9]. Performance differences have been commonly attributed to biological maturation in addition to social, pedagogic and behavioral aspects, as well as the opportunity to engage in extra practice time [10,11,12,13]. Veldhuizen et al., therefore, argue that the RAE is a result of a natural and straightforward relationship between age and performance, because cognitive, behavioral, motor, social and emotional development follow age [13]. Accordingly, a RAE can be expected in circumstances of strict selection procedures, which are common in sports and education [13]. This is also consistent with available research that showed poorer academic performance [1,14,15,16] and worse physical fitness [13,17,18] in children who are born late within a given cohort. Children born early in their cohort, on the other hand, generally enjoy an advantage over their peers and are often considered more talented, which results in a higher representation in sports at school and club level [19,20,21,22]. Similarly, early-born children are more likely to be enrolled in advanced academic programs [16,23]. Relatively older children have also been shown to perform better in physical education (PE) than their younger peers, which was reflected by differences in PE grades [2,24,25,26]. Taken together, the RAE has been observed in explicitly and implicitly competitive situations when participants are stratified by chronological age.

While it can be expected that the RAE disappears as children grow and ultimately reach maturity, there can still be secondary effects. Children born early in a selection year are more likely to have positive experiences, which will result in a higher perceived competence [27]. These children are also more likely to continue with their participation in sports and the additional experiences will further enhance their abilities [28]. As children born early in the year may be perceived as more talented, they may also be given more and better training opportunities [23,29], which will further contribute to enhanced development compared to their non-selected peers. The initial selection, therefore, becomes self-fulfilling, which is known as Pygmalion effect [30]. Relatively younger children, on the other hand, have been shown to display lower self-esteem and greater health problems [31,32]. These children are also more commonly diagnosed with learning difficulties and behavioral problems [33]. Given the importance of physical fitness for general health and development in children, along with its critical role in the promotion of an active lifestyle [34,35,36,37,38], all children should be given equal opportunities to enhance their physical fitness.

Even though the RAE has been shown consistently in education and sport, it is not entirely understood at this time that it is also present in non-selective settings [39]. Further, differences in physical fitness remained after controlling for somatic maturation [17]. Additional research, therefore, is necessary, particularly in less competitive settings, such as PE, in order to reduce the potential discrimination of relatively younger children. The majority of studies also examined adolescents, and there remains limited information on the RAE in children. The present study, therefore, examines differences in physical fitness across birth-quartiles with a 12-month timespan during the elementary-school years.

## 2. Materials and Methods

The study population consisted of 18,168 children between 6 and 11 years of age (mean age: 8.4 ± 0.8 years) that participated in a state-wide, school-based physical fitness assessment, which has been described in detail elsewhere [40]. The study procedures were in accordance with the 2008 declaration of Helsinki and the study protocol has been approved by the Upper Austrian school board as well as the participating schools. Parents provided written consent prior to data collection, and children provided oral assent at the time of measurement.

All measurements were conducted during regular school time in the participating school’s gymnasium by trained personnel. Body weight (kg) and height (cm) were measured according to standard procedures with children wearing sports clothes and being barefoot. Specifically, body weight was measured with an electronic scale (Seca 878 dr, Seca, Hamburg, Germany) to the nearest 0.1 kg, and height was measured with a portable stadiometer (SECA 2013, Seca, Hamburg, Germany) to the nearest 0.5 cm. Body mass index (BMI) was calculated (kg/m^2^) and converted to BMI percentiles (BMIPCT) based on German reference values with the 90th percentile as cutpoint for overweight/obesity [41].

Upon completion of anthropometric measurements, participants completed 8 physical fitness tests that assessed cardiorespiratory endurance, muscular strength and power, speed, agility, flexibility and object control. Cardiorespiratory fitness was assessed via the distance covered during a 6 min run. Muscular strength and power were assessed with a medicine ball push and standing vertical jump. Speed was assessed with a 10 m sprint and a 6 s tapping test. A standardized obstacle course that required a forward roll as well as jumping over and crawling under obstacles, along with directional changes, was used to assess agility. Flexibility was assessed with a stand-and-reach test, and object control was assessed with a throw-and-catch test using a European handball, size 1. Participants had two attempts per test, except for tapping (3 attempts) and the 6 min run (1 attempt), with sufficient recovery time between attempts. The best attempts were used for further analyses. Fitness tests were administered in random order, except for the 6-min run, which was completed at the end of the testing session. Total time for the assessments per participant was between 90 and 120 min.

As tests were administered throughout the school year, chronological age was used (calculated as test date—birthdate) rather than school grade, and participants were grouped into one-year age groups (6.00–6.99 years, 7.00–7.99 years, … 10.00–10.99 years). Within each age group, age-specific quartiles were established based on the decimal value of the chronological age (Quartile 1: 0.00–0.24; Quartile 2: 0.25–0.49; Quartile 3: 0.50–0.74; Quartile 4: 0.75–0.99). Children in quartile 1, therefore, were the youngest participants, and children in quartile 4 were the oldest participants within each age group.

*Statistical analysis.* Descriptive statistics were calculated, and data were checked for normal distribution. Means with standard deviation are reported for interval-scaled data, and nominal and ordinal data are reported as prevalence. In order to examine the RAE in physical fitness in elementary school children, a 5 (age group) × 4 (age-specific quartile) MANCOVA, adjusted for sex, was conducted across the entire sample. In addition, sex-specific 5 × 4 MANOVAs were used to examine potential differences in the RAE between boys and girls. In order to account for the influence of body weight on physical fitness, additional analyses included BMIPCT as covariate. All analyses were performed in SPSS V26.0 software (SPSS Inc., IBM Corp Armonk, New York, NY, USA) with a significance level of alpha < 0.05.

## 3. Results

Across the entire sample (N = 18,168, 51.3% boys), 14.7% of the participants were considered overweight or obese. The prevalence of overweight/obesity was higher in boys compared to girls (15.3% versus 14.0%, *p* = 0.01) and increased from 13.4% in 6- to 7-year-old children to 18.9% in 10- to 11-year-olds. Even though the distribution of children in the quartiles differed within age groups, there was no difference in children in quartiles 1 through 4 across the entire study population (Table 1).

Body weight and height increased significantly across age groups (*p* for trend < 0.01), and there was an increase in BMIPCT with increasing age (*p* for trend = 0.04). Across the entire sample, there was also a significant increase in BMIPCT across age-specific quartiles (*p* for trend = 0.03) (Table 2). Age-group by age-specific quartile interactions for anthropometric characteristics were non-significant.

**Figure 1:**
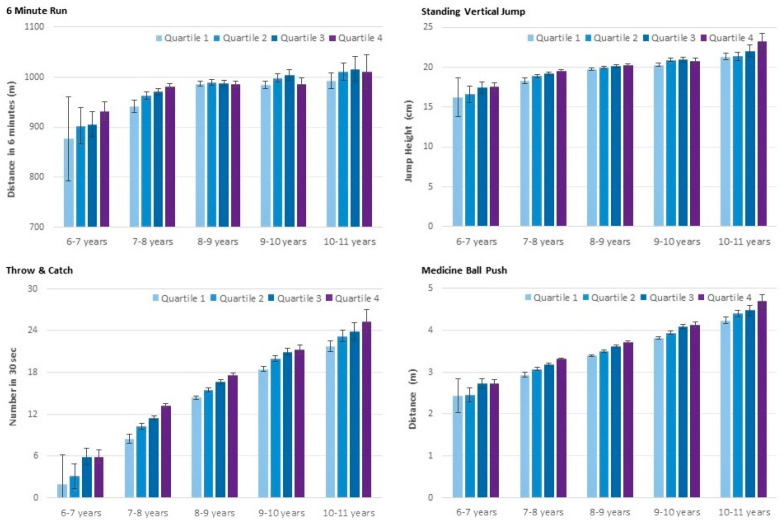
Endurance, muscular power and object control by age-group and age-specific quartile. Values are means, adjusted for sex, with a 95% confidence interval.

**Figure 2:**
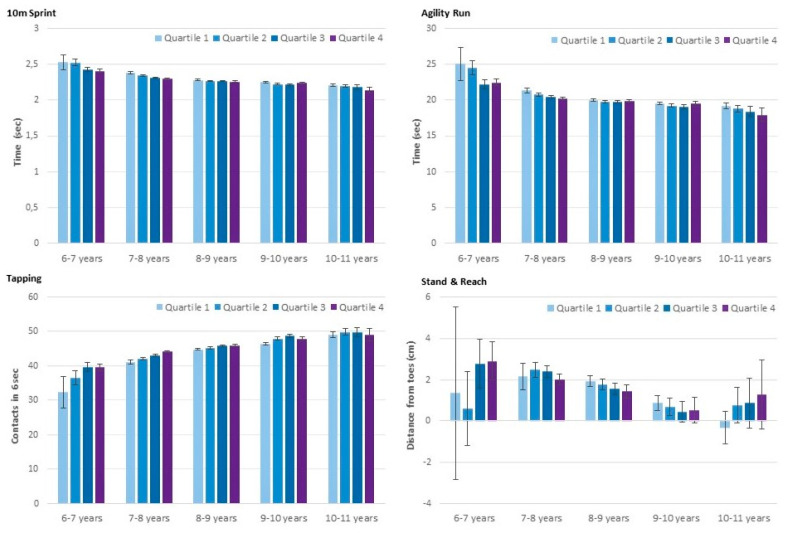
Speed, agility and flexibility by age-group and age-specific quartile. Values are means, adjusted for sex, with a 95% confidence interval.

There was a significant increase in all components of physical fitness across age groups (*p* for trend < 0.01), except for flexibility, wherein a significant decline was observed (*p* for trend < 0.01). In addition, significant differences across age-specific quartiles were observed for strength (medicine-ball push, counter movement jump) speed (tapping, 10 m sprint, agility run) and object control (throw-and-catch) (*p* for trend < 0.01) with better performance in relatively older children. The difference across age-specific quartiles was less pronounced for endurance (*p* for trend = 0.02) and was non-significant for flexibility (Figure 1; Figure 2). In addition, significant age-group by age-specific quartile interaction effects for the 6 min run, counter movement jump, tapping, 10 m sprint and throw-and-catch (*p* < 0.01) revealed a decline in performance differences across quartiles with increasing age in these tests. No significant interaction effects were observed for the medicine-ball push, agility run and stand-and-reach test. These results remained essentially unchanged when additionally controlling for BMIPCT.

Sex-specific analyses revealed similar main effects in boys. Age-group by age-specific quartile interactions were significant for the 6 min run, 10 m sprint, tapping, agility run and throw-and-catch (*p* < 0.01), with a decline in interquartile differences with increasing age. Differences in age-specific quartiles in the 6 min run, for example, were only significant in 7–8-year-old boys, and differences in agility were only significant up to the age of 8 years. No significant interaction effects were observed for strength-related assessments (counter movement jump, medicine-ball push) and flexibility (Table 3). These results remained essentially unchanged after controlling for BMIPCT.

In girls, performance increased significantly across age-specific quartiles for strength (medicine-ball push, counter movement jump) speed (Tapping, 10 m sprint, agility run) and object control (throw-and-catch) (*p* for trend ≤ 0.01). Flexibility also increased across age-specific quartiles (*p* for trend = 0.03), while there was no significant difference across age-specific quartiles in the 6 min run. Significant age-group by age-specific quartile interaction effects were observed for the counter movement jump, tapping, 10 m sprint, agility and flexibility (*p* ≤ 0.01). There was a decline in differences across age-specific quartiles with increasing age in speed-related tasks with significant differences across age-specific quartiles up to the age of 9 years. For flexibility, on the other hand, differences became more pronounced with increasing age and were significant after the age of 9 years. There was also a significant interaction effect for the 6 min run (*p* = 0.02), which was attributed to a less pronounced increase in quartile 4 across age groups, compared to their peers. No significant interaction effects were observed at the medicine-ball push and throw-and-catch (Table 4). These results remained essentially unchanged after controlling for BMIPCT.

## 4. Discussion

The results of the present study show a direct association of age with anthropometric characteristics and various components of physical fitness. Relatively older children within a given age group were taller and heavier than their peers, and a RAE was present for strength and power, speed, agility and object control during the elementary-school years. The RAE was less pronounced for endurance and only significant in boys. Relatively older girls, however, displayed a less pronounced improvement in endurance compared to their peers, which may reflect a greater risk for low physical activity (PA) in this group. There was also a RAE for flexibility in girls, with better performance in older girls compared to their younger peers. Across the entire sample, differences in physical fitness with age groups declined with increasing age for speed and endurance, while they remained relatively stable throughout the elementary-school years for absolute strength. In boys, there was also a decline in the RAE for object control, while interquartile differences remained relatively stable for object control in girls.

Similar results have been shown in previous studies, which showed differences in motor performance and physical fitness in preschoolers [42,43], elementary-school children [18,25] and adolescents [2,18,26]. Even though an age difference of up to 12 months most likely has little or no effect on physical performance in adults, it can result in significant differences in children and adolescents due to rapid changes in growth and development. Accordingly, a RAE has been shown for various components of physical fitness, with better performance in participants born early within a respective age group [13,18,25,39]. Children born earlier are generally taller and have more mass than their younger peers [44]. This provides several advantages, particularly for strength and power but may hinder endurance performance, which could explain the less pronounced RAE at the 6 min run. In addition to differences in anthropometric characteristics, the advantages in physical fitness of relatively older children may be the result of differences in maturation and life-experience, along with potential practice time [13]. Taken together, it has been argued that the RAE can be attributed to the same factors that explain age-related differences between grades, as also shown in this study, and is simply the result of grouping children by chronological age [13,45]. Mainly, in young children, there is an obvious inverse association between age and physical ability, which is most pronounced when comparing a newborn to a one-year-old infant [24]. Even at the time children start elementary school around the age of 6, the age difference between two children in the same class can be up to 16% of total lifetime [46]. Early-born children, therefore, may not only be more mature but also could have had more opportunities to engage in various forms of PA [12]. Throughout the elementary-school years, this lifetime difference is reduced to 10% at the age of 10. It can, therefore, be expected that the RAE is more prominent at younger ages in domains such as motor competence and cognitive achievement [2,5,16] and decreases throughout the elementary-school years.

Differences in physical fitness by relative age, however, have been shown to persist into adolescence [2,26] and remained even after controlling for chronological age and anthropometric characteristics [25]. Accordingly, the RAE cannot simply be attributed to differences in growth and maturation, and there appears to be a complex interaction of individual characteristics with environmental factors and task-related aspects [11]. The previously mentioned advantages of early-born children, for example, may be mistaken for superior abilities and talent at young ages [47]. These children, therefore, are more likely to be selected for sports teams and high-ability groups within and out of school [19], which is also referred to as the maturation-selection hypothesis [4]. This selection bias provides more exposure to PA and sports and better opportunities to enhance physical fitness in relatively older children. Even in less selective settings, such as PE, earlier-born children have an advantage [2,24] as PE assessments are often product-focused [48]. Accordingly, various studies reported better grades in PE in relatively older children [2,19,23,49]. Such positive experiences further enhance enjoyment, confidence and self-esteem that encourage the engagement in additional training and practice, along with more favorable attitudes towards PA [23,50]. Relatively younger children, on the other hand, are at an increased risk for frustrating experiences in sports or PE and may be mistakenly perceived as less talented [4]. Relatively younger children, therefore, are more likely to develop negative attitudes towards PA and sports, which may lead to withdrawal from various forms of PA. Low locomotor skill competence, for example, doubled the risk for insufficient PA in girls, and poor object control skills were associated with not meeting PA recommendations in boys [51]. The difference in engagement in sports and PA subsequently limits the chance to reduce the RAE even though differences in biological development become less pronounced with increasing age. RAEs observed during childhood, therefore, may persist into adolescence, particularly in areas that rely on physical competences, including PE [19].

Given the long-term consequences of an initially age-associated difference in physical fitness, it is critical to enhance the awareness of teachers, coaches and education policymakers on the presence of a RAE. In fact, it has been argued that the RAE is compounded by current age-grouping and selection strategies in schools and outside the classroom due to the assumption that children within the same age group have similar abilities and learner characteristics [2,19]. Even in non-selective settings, such as PE, it is, therefore, important to find strategies that address the variability in performance due to differences in relative age [49]. In club sports, there have been attempts to classify children and adolescents based on their biological age, rather than their chronological age [52], but this may be difficult in an educational setting. It may, however, be possible to account for differences by age within a given age group by considering age bands (e.g., age quartiles). Another option could be a test administration at different time points, which puts the participants at a similar age at their assessment (e.g., later assessment in relatively younger children). Further, the evaluation of product-oriented fitness tests should be based on individual progress, rather than comparing individual performances across children within a specific grade. PE teachers should also consider the different abilities of their students and account for them when preparing their lessons regarding volume and intensity (e.g., allow for variability in number of sets, repetitions, task difficulty, …). As motor development is a major objective of PE [53,54], it is critical to provide inclusive and meaningful opportunities for all children [55]. Considering individual differences in motor development and providing adequate movement experiences at various levels may allow younger students to reach similar levels or even surpass their older peers at a later stage. Another approach may be a stronger focus on participation rather than performance, which could enhance motivation and attitudes towards PE that may transfer into active leisure time choices that stimulate the development of physical fitness and motor competence.

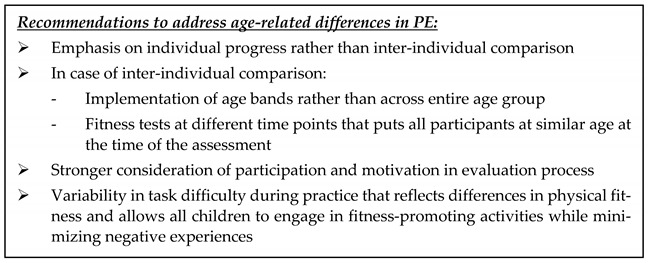



The potential benefits of such alterations in PE programs, however, cannot be examined by the present study due to its cross-sectional nature. It should also be considered that there was no additional data on correlates of physical fitness, such as participation in sports, leisure time PA and socio-economic background, as well as biological maturation. Potential differences in motivation may also have influenced individual performance during the fitness tests. The large sample size, along with the administration of multiple assessments by trained personnel that cover various aspects of physical fitness, on the other hand, is a considerable strength of this study, which provides plausible evidence of a RAE in Austrian elementary-school children.

## 5. Conclusions

The available evidence shows that a RAE is commonly observed when participants are grouped according to chronological age even though biological maturation and anthropometric development, as well as experience and practice opportunities, vary considerably with age. Despite these differences, every child should be given opportunities to engage successfully in PA and sports without discrimination [56]. Given the wealth of descriptive research on long-term consequences of the RAE, continued efforts to reduce biases due to RAE in various settings, including PE and sports, are warranted. Schools, for example, can reconsider their approach in teaching and evaluating students’ performance, as well as their approach to selection and provision for school sports. Similarly, coaches need to consider that relatively younger children may have the same potential for high performance at a later stage when evaluating their performance [25]. All children should be given opportunities to enhance their physical fitness and enjoy participation in various forms of PA, independent of their current abilities, in order to facilitate an active and healthy lifestyle beyond childhood and adolescence.

## Figures and Tables

**Table 1:** Distribution of participants across age groups and age-specific quartiles.

	Quartile 1 N (% Boys)	Quartile 2 N (% Boys)	Quartile 3 N (% Boys)	Quartile 4 N (% Boys)
6–7 years	9 (55.6%)	49 (57.1%)	111 (50.5%)	183 (59.0%)
7–8 years	389 (51.4 %)	1128 (46.6%)	1850 (48.7%)	2508 (49.9%)
8–9 years	2653 (49.3%)	2242 (50.7%)	1820 (52.6%)	1441 (53.7%)
9–10 years	1233 (53.0%)	900 (56.8%)	600 (54.5%)	411 (59.9%)
10–11 years	259 (54.4%)	217 (48.4%)	108 (50.0%)	57 (64.9%)
Total Sample	4543 (50.8%)	4536 (50.9%)	4489 (51.1%)	4600 (52.5%)

Values are the number of participants with the percentage of boys.

**Table 2:** Anthropometric characteristics by age group and age-specific quartiles.

Age		Quartile 1	Quartile 2	Quartile 3	Quartile 4	TOTAL
**6–7 years**	Height (cm)	121.3 ± 7.3	121.4 ± 5.8	122.0 ± 5.0	123.6 ± 5.1	122.7 ± 5.3
	Weight (kg)	23.4 ± 5.7	23.9 ± 4.9	24.2 ± 4.5	24.5 ± 4.5	24.3 ±4.6
	BMIPCT	48.3 ± 31.7	54.2 ± 25.6	53.5 ± 30.4	49.3 ± 30.4	51.3 ±29.8
**7–8 years**	Height (cm) ^1^	125.7 ± 5.6	126.8 ± 5.4	128.4 ± 5.4	130.0 ± 5.5	128.6 ± 5.7
	Weight (kg) ^1^	25.7 ± 5.2	26.3 ± 5.3	27.0 ± 5.1	28.1 ± 5.5	27.2 ± 5.4
	BMIPCT	50.6 ± 28.5	50.0 ± 28.7	50.4 ± 28.5	51.4 ± 29.0	50.8 ± 28.7
**8–9 years**	Height (cm) ^1^	130.9 ± 5.6	132.0 ± 5.6	133.4 ± 5.8	134.7 ± 6.2	132.5 ± 5.9
	Weight (kg) ^1^	28.6 ± 5.9	29.4 ± 6.2	30.5 ± 6.6	31.7 ± 7.5	29.8 ± 6.6
	BMIPCT ^1^	50.3 ± 29.3	51.3 ± 29.3	52.5 ± 30.2	53.5 ± 30.2	51.6 ± 29.7
**9–10 years**	Height (cm) ^1^	136.0 ± 6.2	137.2 ± 6.4	138.8 ± 6.5	139.7 ± 6.5	137.4 ± 6.5
	Weight (kg) ^1^	32.5 ± 7.7	33.4 ± 8.0	34.4 ± 8.2	36.1 ± 9.1	33.6 ± 8.2
	BMIPCT ^1^	53.1 ± 31.0	53.6 ± 31.2	53.4 ± 31.4	58.0 ± 31.2	53.9 ± 31.2
**10–11 years**	Height (cm) ^1^	141.0 ± 6.3	142.1 ± 6.5	143.8 ± 6.8	144.3 ± 6.7	142.1 ±6.6
	Weight (kg) ^1^	36.4 ± 9.4	36.7 ± 8.4	38.7 ± 9.0	39.2 ± 11.7	37.1 ± 9.3
	BMIPCT	53.6 ± 32.0	53.4 ± 31.5	55.1 ± 32.1	53.7 ± 32.4	53.8 ± 31.8

Values are means ± SD. ^1^ *p* for trend across quartiles < 0.01.

**Table 3:** Physical fitness by age-group and age-specific quartile in boys.

		Quartile 1	Quartile 2	Quartile 3	Quartile 4	TOTAL
**6–7 years**	6 Min Run (m)	880 ± 129	920 ± 136	919 ± 127	946 ± 124	933 ± 127
	Ball Push (cm) ^1^	246 ± 49	249 ± 45	291 ± 47	291 ± 0.6	284 ± 57
	Vertical Jump (cm)	17.4 ± 5.2	17.0 ± 2.9	17.7 ± 3.3	17.4 ± 3.3	17.4 ± 3.3
	Sprint (sec) ^1^	2.57 ± 0.28	2.50 ± 0.16	2.39 ± 0.15	2.40 ± 0.18	2.42 ± 0.17
	Tapping (contacts) ^2^	32.8 ± 8.7	38.0 ± 5.1	40.9 ± 7.4	41.0 ± 7.0	40.4 ± 7.0
	Agility (sec) ^1^	25.8 ± 7.4	24.4 ± 4.0	21.9 ± 3.3	22.5 ± 4.1	22.7 ± 4.1
	Stand-Reach (cm) *	2.8 ± 7.1	0.3 ± 5.6	1.8 ± 5.5	1.3 ± 5.2	1.4 ± 5.4
	Throw & Catch (#)	3.8 ± 6.9	4.6 ± 4.4	7.1 ± 5.9	7.6 ± 6.3	7.0 ± 6.0
**7–8 years**	6 Min Run (m) ^2^	966 ± 129	1002 ± 127	1004 ± 120	1015 ± 130	1006 ± 127
	Ball Push (cm) ^2^	316 ± 51	329 ± 54	338 ± 57	355 ± 59	342 ± 58
	Vertical Jump (cm) ^2^	18.5 ± 3.3	19.4 ± 3.3	19.7 ± 3.4	20.0 ± 3.6	19.7 ± 3.5
	Sprint (sec) ^2^	2.34 ± 0.16	2.30 ± 0.15	2.28 ± 0.15	2.27 ± 0.16	2.28 ± 0.16
	Tapping (contacts) ^2^	42.8 ± 6.4	44.0 ± 6.3	45.0 ± 6.4	46.0 ± 6.5	45.1 ± 6.5
	Agility (sec) ^2^	20.7 ± 3.2	20.2 ± 3.0	19.9 ± 3.2	19.7 ± 3.4	19.9 ± 3.3
	Stand-Reach (cm) *	1.0 ± 6.0	1.2 ± 5.7	0.9 ± 5.5	0.6 ± 6.2	0.8 ± 5.9
	Throw & Catch (#) ^2^	10.7 ± 6.0	12.9 ± 69	13.5 ± 6.5	15.8 ± 6.4	14.2 ± 6.7
**8–9 years**	6 Min Run (m)	1022 ± 138	1023 ± 132	1021 ± 131	1015 ± 144	1021 ± 136
	Ball Push (cm) ^2^	361 ± 60	372 ± 62	384 ± 65	395 ± 69	376 ± 65
	Vertical Jump (cm) ^1^	20.3 ± 3.8	20.4 ± 3.8	20.8 ± 3.9	20.7 ± 3.9	20.5 ± 3.9
	Sprint (sec) ^2^	2.25 ± 0.16	2.24 ± 0.16	2.23 ± 0.17	2.23 ± 0.16	2.24 ± 0.16
	Tapping (contacts) ^2^	46.5 ± 6.4	47.0 ± 6.9	47.6 ± 7.3	47.4 ± 7.3	47.0 ± 6.9
	Agility (sec)	19.5 ± 3.5	19.2 ± 3.2	19.3 ± 3.8	19.6 ± 4.0	19.4 ± 3.6
	Stand-Reach (cm) *^,1^	0.3 ± 6.1	0.4 ± 6.3	0.0 ± 6.4	−0.2 ± 6.8	0.1 ± 6.4
	Throw & Catch (#) ^2^	16.6 ± 6.5	17.4 ± 6.6	18.7 ± 6.6	19.2 ± 6.6	17.8 ± 6.7
**9–10 years**	6 Min Run (m)	1012 ± 154	1024 ± 158	1028 ± 156	1017 ± 145	1019 ± 154
	Ball Push (cm) ^2^	404 ± 91	413 ± 70	434 ± 71	440 ± 66	417 ± 80
	Vertical Jump (cm) ^2^	20.8 ± 4.3	21.4 ± 4.5	21.5 ± 4.1	21.6 ± 4.4	21.2 ± 4.3
	Sprint (sec) ^1^	2.22 ± 0.17	2.20 ± 0.17	2.19 ± 0.16	2.20 ± 0.18	2.21 ± 0.17
	Tapping (contacts) ^2^	48.0 ± 7.3	49.5 ± 7.3	50.5 ± 7.0	50.1 ± 7.8	49.2 ± 7.4
	Agility (sec)	19.1 ± 4.1	18.8 ± 3.9	18.7 ± 4.0	18.9 ± 4.2	18.9 ± 4.0
	Stand-Reach (cm) *	−0.8 ± 6.7	−1.3 ± 7.0	−1.1 ± 7.2	−0.5 ± 6.9	−1.0 ± 7.0
	Throw & Catch (#) ^2^	20.0 ± 6.7	21.5 ± 6.5	22.5 ± 6.3	23.1 ± 6.3	21.3 ± 6.6
**10–11 years**	6 Min Run (m)	1009 ± 161	1039 ± 152	1028 ± 148	1050 ± 144	1026 ± 155
	Ball Push (cm) ^1^	444 ± 73	472 ± 81	464 ± 99	480 ± 84	460 ± 82
	Vertical Jump (cm) ^1^	21.8 ± 4.6	21.9 ± 4.1	22.0 ± 4.9	23.8 ± 5.0	22.1 ± 4.6
	Sprint (sec) ^1^	2.19 ± 0.19	2.16 ± 0.17	2.17 ± 0.17	2.12 ± 0.15	2.17 ± 0.18
	Tapping (contacts)	50.5 ± 8.7	51.8 ± 7.9	49.9 ± 9.0	50.7 ± 10.2	50.8 ± 8.7
	Agility (sec)	18.6 ± 3.5	18.1 ± 4.2	18.4 ± 4.1	17.3 ± 3.5	18.3 ± 3.8
	Stand-Reach (cm) *	−1.9 ± 7.4	−0.9 ± 7.6	−1.9 ± 6.3	−1.2 ± 7.9	−1.5 ± 7.3
	Throw & Catch (#) ^1^	22.7 ± 7.3	25.2 ± 6.7	24.2 ± 7.4	25.9 ± 6.4	24.1 ± 7.1

Values are means ± SD. # number of repetitions; * positive values indicate reaching beyond the toes, and negative values indicate not reaching toes; ^1^ *p* for trend across quartiles < 0.05; ^2^ *p* for trend across quartiles < 0.01.

**Table 4:** Physical fitness by age-group and age-specific quartile in girls.

		Quartile 1	Quartile 2	Quartile 3	Quartile 4	TOTAL
**6–7 years**	6 Min Run (m)	878 ± 91.8	887 ± 109	891 ± 124	924 ± 111	906 ± 115
	Ball Push (cm)	245 ± 29	246 ± 36	251 ± 43	252 ± 46	251 ± 43
	Vertical Jump (cm) ^1^	14.8 ± 2.5	16.2 ± 2.4	17.2 ± 3.5	17.9 ± 3.1	17.4 ± 3.2
	Sprint (sec)	2.47 ± 0.08	2.54 ± 0.17	2.46 ± 0.16	2.41 ± 0.16	2.45 ± 0.17
	Tapping (contacts)	32.0 ± 5.4	34.8 ± 5.4	38.2 ± 7.0	38.1 ± 740	37.5 ± 7.1
	Agility (sec)	24.0 ± 2.6	24.6 ± 4.7	22.6 ± 3.2	22.3 ± 3.2	22.8 ± 3.5
	Stand-Reach (cm) *^,1^	−0.8 ± 9.0	0.5 ± 6.2	3.8 ± 5.3	4.6 ± 6.9	3.6 ± 6.4
	Throw & Catch (#) ^1^	0.0 ± 0.0	1.7 ± 1.9	4.6 ± 5.1	4.2 ± 4.7	3.9 ± 4.6
**7–8 years**	6 Min Run (m) ^2^	916 ± 113	923 ± 117	934 ± 114	946 ± 117	936 ± 116
	Ball Push (cm) ^2^	268 ± 49	283 ± 50	298 ± 53	306 ± 51	296 ± 53
	Vertical Jump (cm) ^2^	18.1 ± 3.2	18.4 ± 3.3	18.8 ± 3.2	19.1 ± 3.4	18.8 ± 3.3
	Sprint (sec) ^2^	2.42 ± 0.18	2.38 ± 0.16	2.34 ± 0.16	2.32 ± 0.15	2.35 ± 0.16
	Tapping (contacts) ^2^	39.2 ± 6.7	40.1 ± 6.4	41.0 ± 6.5	42.1 ± 6.5	41.1 ± 6.6
	Agility (sec) ^2^	22.0 ± 3.6	21.4 ± 3.1	20.9 ± 3.1	20.7 ± 3.3	21.0 ± 3.2
	Stand-Reach (cm) *	3.4 ± 5.5	3.9 ± 6.2	4.0 ± 6.2	3.5 ± 6.3	3.7 ± 6.2
	Throw & Catch (#) ^2^	6.2 ± 5.9	7.6 ± 5.7	9.2 ± 6.1	10.5 ± 6.3	9.3 ± 6.2
**8–9 years**	6 Min Run (m)	949 ± 116	954 ± 120	952 ± 120	955 ± 127	952 ± 129
	Ball Push (cm) ^2^	316 ± 54	326 ± 55	337 ± 59	346 ± 60	328 ± 58
	Vertical Jump (cm) ^2^	19.2 ± 3.4	19.5 ± 3.6	19.6 ± 3.7	19.8 ± 3.8	19.5 ± 3.6
	Sprint (sec) ^2^	2.31 ± 0.16	2.30 ± 0.16	2.29 ± 0.18	2.29 ± 0.18	2.30 ± 0.17
	Tapping (contacts) ^2^	42.9 ± 6.9	43.2 ± 6.9	43.9 ± 7.4	44.5 ± 7.5	43.4 ± 7.2
	Agility (sec) ^1^	20.5 ± 3.2	20.3 ± 3.4	20.3 ± 3.7	20.2 ± 3.8	20.3 ± 3.5
	Stand-Reach (cm) *	3.7 ± 6.5	3.2 ± 6.4	3.2 ± 6.6	3.2 ± 6.6	3.4 ± 6.5
	Throw & Catch (#) ^2^	12.0 ± 6.3	13.4 ± 6.2	14.5 ± 6.6	15.8 ± 6.7	13.6 ± 6.5
**9–10 years**	6 Min Run (m)	957 ± 122	973 ± 117	980 ± 127	951 ± 129	965 ± 123
	Ball Push (cm) ^2^	357 ± 63	373 ± 64	383 ± 70	382 ± 71	370 ± 66
	Vertical Jump (cm)	19.8 ± 4.0	20.4 ± 4.0	20.3 ± 4.2	19.8 ± 4.0	20.1 ± 4.0
	Sprint (sec)	2.28 ± 0.16	2.25 ± 0.16	2.24 ± 0.16	2.28 ± 0.18	2.26 ± 0.16
	Tapping (contacts)	44.5 ± 8.0	46.2 ± 7.3	46.7 ± 8.0	45.2 ± 8.3	45.5 ± 7.9
	Agility (sec)	19.9 ± 3.6	19.6 ± 3.9	19.4 ± 3.2	20.3 ± 3.9	19.8 ± 3.7
	Stand-Reach (cm) *^,1^	2.6 ± 6.8	2.9 ± 7.2	2.1 ± 7.0	1.4 ± 7.0	2.5 ± 7.0
	Throw & Catch (#) ^2^	16.9 ± 6.6	18.4 ± 6.6	19.4 ± 6.3	19.4 ± 6.6	18.1 ± 6.6
**10–11 years**	6 Min Run (m)	978 ± 151	980 ± 137	1002 ± 136	963 ± 131	982 ± 142
	Ball Push (cm) ^2^	402 ± 62	406 ± 73	430 ± 66	466 ± 84	413 ± 70
	Vertical Jump (cm) ^1^	20.8 ± 4.6	20.8 ± 4.4	22.1 ± 4.8	22.6 ± 5.2	21.2 ± 4.6
	Sprint (sec)	2.23 ± 0.16	2.23 ± 0.19	2.19 ± 0.16	2.16 ± 0.17	2.22 ± 0.19
	Tapping (contacts)	47.6 ± 8.3	47.8 ± 8.7	49.3 ± 8.4	47.2 ± 7.1	48.0 ± 8.4
	Agility (sec)	19.7 ± 5.4	19.5 ± 4.5	18.4 ± 3.2	18.9 ± 3.0	19.4 ± 4.6
	Stand-Reach (cm) *^,1^	1.3 ± 6.4	2.5 ± 6.5	3.7 ± 6.4	4.7 ± 7.0	2.4 ± 6.5
	Throw & Catch (#) ^2^	20.7 ± 6.2	21.0 ± 6.5	23.4 ± 25.8	25.8 ± 4.6	21.7 ± 6.3

Values are means ± SD. # number of repetitions; * positive values indicate reaching beyond the toes, and negative values indicate not reaching toes; ^1^ *p* for trend across quartiles < 0.05; ^2^ *p* for trend across quartiles < 0.01.

## Data Availability

The raw data supporting the conclusions of this article will be made available by the authors, without undue reservation.
